# Doppler flowmetry as a tool of predictive, preventive and personalised dentistry

**DOI:** 10.1186/1878-5085-4-21

**Published:** 2013-08-28

**Authors:** Liudmila Yu Orekhova, Anna A Barmasheva

**Affiliations:** 1 Therapeutic Dentistry Department, I.P. Pavlov Saint-Petersburg State Medical University, 6/8 Lev Tolstoy Street, Saint Petersburg 197022, Russia; 2 City Periodontal Center ‘PAKS’, 27 Dobrolubova Prospect, Saint-Petersburg 197198, Russia

**Keywords:** Predictive periodontology, Preventive periodontology, Personalised periodontology, Microcirculation, Laser Doppler flowmetry, Ultrasonic Doppler flowmetry, Gingival blood flow, Pulpal blood flow

## Abstract

Periodontal lesions are considered a major problem in the global burden of oral diseases due to their high frequency and negative impact on quality of life. Periodontal inflammation is accomplished by a breakdown of microcirculatory function. Early detection of gingival microvessel dysfunction helps diagnose and prevent the progression of initial periodontal pathology. Doppler flowmetry is a useful tool in the diagnosis, monitoring, prognosis and management of periodontal patients which allows access not only of gingival blood flow but also of pulpal microcirculation. Doppler flowmeters might help to realise the ultimate target of predictive, preventive and personalised periodontology tailored with respect to the particular patient. This article highlights the main working principles of laser Doppler flowmeters and the ultrasonic Doppler flowmeters. The advances in blood flow measurement by ultrasonic flowmetry are discussed.

## Review

### Periodontitis and microcirculatory dysfunction

Periodontitis is a highly prevalent chronic inflammatory disorder with a negative impact on the quality of life affecting 30% to 40% of the population over 35 years old [1]. It involves the breakdown of tooth-supporting tissues and subsequent loss of teeth and is considered a major problem in the global burden of oral diseases [2]. It has been estimated that in developed countries, about 50% of the adult population has gingivitis in approximately three or four teeth at any given time and 30% has periodontitis (presence of three or more teeth with pockets of ≥4 mm) [3,4].

The main causes of gingival inflammation are an ecological imbalance between the oral microbial biofilm and an impaired host inflammatory response [5-7]. Periodontal inflammation often leads to superficial ulcers on the gingival sulcus, where blood capillaries are exposed to microbial biofilms [8]. Periodontal pathogens are translocated and released from the sulcus into the bloodstream leading to breakdown of microcirculatory function. On the other hand, dysfunction of microcirculation may impair tissue perfusion and result in organ dysfunction [9,10]. Inflammatory mediators that increase vascular permeability in microvessels with adherens junctions exert this effect by disrupting junctional complex assembly via phosphorylation, internalisation, and/or degradation of junctional molecules [11]. Gingival inflammation results in increased number of capillary loops, enlargement of the vessel size and slowing of blood flow, and limitation of the afferent blood vessels. Research indicated an interaction between gingival blood flow and gingival health [12]. It is considered that changes of the vascular morphology due to inflammation are associated with blood flow changes. These changes may be the first sign to predict the onset of pathological events in the gingival tissue, and dysfunction of the gingival blood flow may serve as a prognostic marker [13]. This highlights a useful role of different devices assessing oral blood flow in research and in routine practice.

### Evaluation of the blood blow by Doppler flowmeters

Today various methods are available for the investigation of blood supply in clinics, some of which are excellent in determining the presence and severity of arterial and venous disorders. However, the main drawback of these methods is the lack of any information on microvascular status of the diseased areas. This is particularly true for skin circulation, which has a rather complex vascular network with nutritional and thermoregulatory vessels, arteriovenous shunts, etc. [14]. For a long period, the most useful methods for clinical evaluation of the skin capillary circulation were considered to be vital capillary microscopy and dynamic capillaroscopy [14-16], which were also used to study blood flow in the oral mucosa [17,18] and gingiva [19,20].

Since the invention of Doppler flowmeters, useful information relating to the function and viability of an organ became available to scientists and clinical practitioners. The Doppler flowmeters are non-invasive and relatively simple instruments that have found enormous applications in research as well in clinics. The detection of blood flow by Doppler flowmeters is based on a Doppler effect. The Doppler effect or Doppler shift, named after the physicist Christian Doppler who proposed it in 1842, is the shift in frequency and wavelength of waves which results from a source moving with respect to the medium, a receiver moving with respect to the medium or even a moving medium [21]. It is possible to measure microcirculation by Doppler flowmeters because the moving particles that are analysed in the tissue are mainly erythrocytes. Skin flow is most largely investigated by Doppler flowmetry. Nevertheless, data obtained from the skin cannot be extrapolated to other tissues because every tissue has its own microcirculatory characteristics [22].

### Laser Doppler flowmetry

Laser Doppler flowmetry (LDF) or laser Doppler velocimetry has been extensively used in medical and dental research. LDF is a non-invasive technique widely used in haemodynamic research for assessing microvascular blood flow, in particular, to partially quantify blood flow in human tissues such as the skin [23]. This method might also be applicable in longitudinal studies if standardised procedures are used to improve reproducibility [24].

LDF was firstly demonstrated by Maiman [25]. Then a method of measuring the velocity of particles in solution using the Doppler frequency shift of backscattered light was suggested [26]. Over the years, this technique was used to measure the velocity of red blood cells in a glass-tube flow model [27]. More recently, it was applied in the investigation of blood perfusion in the undisturbed microcirculation [28]. Several studies found a correlation between laser Doppler flowmetry and tissue viability [22,29]. It was estimated that the sensitivity of the method is 85% [22].

The LDF evaluates blood flow in capillaries that are close to the skin surface and the flow in the underlying arterioles and venules involved in the regulation of skin temperature [30]. It works with a low-power light from a monochromatic (single-wavelength) stable laser that is scattered by moving red blood cells and, as a consequence, has its frequency shifted. The laser beam has a penetration depth of approximately 1 mm in a hemispherical fashion [31], the capillary diameters are 10 μm and the velocity spectrum measurement is typically 0.01 to 10 mm/s [30]. The light is partly absorbed and partly reflected. Moving particles, mainly erythrocytes, cause a Doppler shift in the reflected light [32,33]. This change in frequency is converted to a laser Doppler flow signal, which is linearly related to microcirculatory blood flow. A blood flow measurement is based on the processing of photodetected frequency-broadened light and laser light scattered from static tissue. For this purpose, there are two optical fibres in laser Doppler probes: one is used to deliver light to the tissues, and the other is used to collect the scattered light. The signal is commonly recorded as the concentration and velocity (flux) of cells using a term ‘perfusion units’ (PU) [34,35] where 2.5 V of blood flow is equivalent to 250 PU [36,37].

### Laser Doppler flowmetry in clinical dentistry

LDF technique was first described in dental literature in 1986 by Gazelius et al. [38]. Of importance, the probe during dental investigation needs to be completely still to make a record of the Doppler shift. That is why it is largely used as a stabilising splint made of polyvinyl siloxane or acrylic [34]. The reproducibility of LDF measurements taken after vasodilation by heat provocation is greater compared to that of basal flow. That is why the use of thermoprobes is highly recommended [24]. Besides, repeat measurements should be taken at the same time of day. In longitudinal studies, LDF has an acceptable reproducibility if the data are obtained from subjects serving as their own controls [24].

There are numerous applications of LDF in dental research as well as in dental practice, in particular, this method is used for the investigation of microcirculation and vitality of the dental pulp [37,39], periodontal ligament [40], gingival or sulcular blood flow in health and disease [41-44], the effect of orthodontic treatment [45,46], the injection of vasoconstrictive anaesthetics [47] in blood flow or assessing bone vascularity in the human mandible during implant insertion [48]. Some of these applications need to be discussed in more detail due to their clinical importance.

### Laser Doppler flowmetry in periodontics

LDF provides data on the blood flow of the marginal gingiva at different dental regions. However, there exists a difference in blood supply of marginal gingiva of the upper and lower jaws [49] as well as a difference between blood flow at the premolar and molar sites and front teeth sites [50]. A statistically significant difference was also demonstrated between blood supply in the maxillary and the mandibular anterior gingiva in the interdental gingiva, attached gingiva and alveolar mucosa [51]. The difference was significant for the mandibular anterior gingiva only in the alveolar mucosa region [51].

Scattering of the surrounding tissue as well as morphological characteristics such as gingival thickness, in particular, periodontal biotypes, might influence LDF variability [13,52]. Age readings as well as the epithelial thickness also affect the gingival vasculature, decreasing LDF readings [53]. Mechanical stimulation of the gingiva, for example, during tooth brushing, significantly increases gingival blood flow in the papillary gingiva of healthy individuals [54].

Marginal blood flow can also be affected by individual characteristics of the restorations or plaque accumulation index [55]. Vag and Fazekas investigated the effects of crown margin on gingival health and found a correlation between gingival index and LDF results [56]. al-Wahadni et al. found higher gingivitis levels on resin-bonded fixed bridges caused by plaque accumulation [57]. Subgingival restoration margins might bring forth an additional inflammatory effect on the gingival tissue resulting in higher blood flow values of test sites [11,58]. Nevertheless, LDF have limited diagnostic value when it comes to clinical performance of fixed prosthesis [12].

Gingival microcirculation exhibits a dramatic, dynamic change in response to the development and progression of gingivitis. However, studies indicate controversies in an existing relationship between plaque accumulation, gingival inflammation and tissue microcirculation. The growth of blood flow in an inflamed gingiva in comparison with a healthy gingiva was demonstrated in several animal [42,59,60] and clinical studies [13,51,61-63]. According to Kerdvongbundit et al., inflammation alters the microcirculatory and micromorphologic dynamics of the human gingiva before and after conventional treatment (scaling and root planning) [62,63]; however, blood flow returned to normal after treatment and remained stable for 3 months post-treatment. It is contrary to what Matheny et al. reported where there was a decrease of blood flow in the inflamed gingiva and an increase in the number of superficial vessels [64]. Other clinical studies found a positive correlation between LDF results and gingival inflammation or bleeding on probing [43,56,65].

LDF is an unbiased non-invasive method of monitoring the response to periodontal therapy [65]. It is adequate for recording changes in gingival blood flow following periodontal surgery presenting different patterns of microvascular blood flow alterations during the wound-healing period [66]. The gingival blood flow decreases immediately following anaesthesia and remains in lower values compared to baseline immediately following operation [52]. Comparison of the gingival blood flow responses following simplified papilla preservation technique versus modified Widman flap indicated that the first method may be associated with faster recovery of the gingival blood flow post-operatively [67].

The LDF method also proved that smoking alters gingival blood flow. In young people, a significant, immediate increase in gingival blood flow was observed during smoking that afterwards returned toward baseline within 10 min [68]. It is speculated that small repeated vasoconstrictive attacks due to cigarette smoking might in the long run contribute to gingival vascular dysfunction and periodontal disease [69]. However, Palmer et al. do not seem to support the theory that tobacco smoking causes localised vasoconstriction in the periodontal tissues in humans [70]. This may be due to elevation in blood pressure induced by smoking, which overcomes any vasoconstrictive effects of smoking [69]. Mullally proposed that LDF in periodontics is only applicable in the measurement of acute changes in blood flow [71]. However, it was shown that smoking causes an acute increase in relative blood flow in the forehead skin in light smokers compared to heavy smokers, suggesting a potential induction of tolerance in regular users of tobacco [72]. Moreover, gingival blood vessels in smokers with healthy gingival conditions respond differently to administration of an anaesthetic containing a vasoconstrictor in comparison with those of non-smokers [47].

Changes in gingival blood flow after orthodontic force application were also studied by the LDF technique. It was estimated that this change correlated to the degree of force applied to displace the teeth, although individual responses to the same degree of force varied in dependence on the degree of tooth displacement [73] and the size of the interdental space [73,74]. The regression coefficient of decreased blood flow to the percentage of tooth displacement was significantly higher in young subjects than in adults [74]. Barta et al. showed that the application of a force of 75 g to the maxillary canine in an ectopic position resulted in a decrease in gingival blood flow up to 50%, but it returned toward the baseline after a few months [75].

### Laser Doppler flowmetry in endodontics

LDF is the objective method to assess pulpal blood flow, even though it is not commonly used in clinical settings due to its expense and the time involved. This technique has been successfully utilised for estimating pulpal vitality in adults and children, differential diagnosis of apical radiolucencies (on the basis of pulp vitality), examining the reactions to pharmacological agents or electrical and thermal stimulation and monitoring of pulpal responses to orthodontic procedures and traumatic injuries [76,77].

It was proven that LDF better indicates pulpal vitality than traditional vitality tests [38,78-80]. LDF is more reliable as compared to pulseoximetry and electric pulp test [81] or compared to usual ‘sensitivity testing’. LDF scores 1.0 for sensitivity and 1.0 for specificity regarding vital or non-vital pulp tissue [82].

Many studies showed that blood circulation and not innervation is the most accurate determinant in assessing pulp vitality, as it provides an objective differentiation between necrotic and vital pulp tissue [77,83]. It was shown that LDF clinical findings were different for primary incisor teeth prior to pulpotomy or extraction in comparison with the same teeth at an earlier time [84].

The method is considered to be highly efficient in assessing pulpal vitality in healthy and traumatised teeth. LDF proved to be the most effective and early indicator for revascularisation of the pulp (3 weeks) [85]. Yanpiset et al. correlated clinical LDF findings to histological findings in the same teeth [86]. They showed that LDF was effective in determining revascularisation in dental pulp and stated that method could provide information about healing progress in the pulps earlier because the flux value increased significantly from week 2 to week 4 in the revascularising pulps.

The LDF test might be useful for showing signs of adverse outcomes in luxated teeth. It was suggested to use predictive modelling for identifying ‘at-risk’ teeth early after the trauma and initiation of their treatment in advance, reducing the risk of the tooth being lost because of pulpal necrosis and infection [87-89]. Besides that, LDF can detect revascularisation in case of avulsion after a few weeks, in advance of other more traditional clinical tests [85].

Pulpal responses to orthodontic forces or orthopaedic forces created by rapid maxillary expansion have been investigated by LDF [90-95]. McDonald and Pitt Ford found that human pulpal blood flow was decreased when continuous light tipping forces were applied to a maxillary canine [90]. Of the possible force factors that can be applied to teeth during orthodontic treatment, intrusion is proposed to have the greatest impact on the apical region [91]. Several studies observed an obvious reduction of pulpal blood flow due to orthodontic tooth movement and marked histologic changes in the pulp as a result of intrusive forces [93,96-99]. Barwick and Ramsay evaluated the effect of a 4-min application of intrusive orthodontic force on human pulpal blood flow and concluded that pulpal blood flow was not altered during the application of a brief intrusive orthodontic force [91]. Among patients who undergo a segmental maxillary osteotomy or Le fort I osteotomy, significant reduction in pulpal sensibility has been noted in teeth in the osteotomised segment or maxilla [100].

### Limitations and drawbacks of laser Doppler flowmetry

The existing controversies might be on several limitations of the technique. A major LDF drawback is that it only detects movement of erythrocytes in a small volume of tissue that is about 1 mm^3^. That is why it cannot analyse different variables, in particular, the flow in individual microvessels, the number of vessels with an active flow and changes in vessel diameter [64]. The other disadvantage emerging from this is a poor reproducibility of the results because a minimal displacement of the probe will lead to a change in the investigated area because of the density of the vascular network of the gum [46].

The artefacts caused by tissue motion in relation to the probe might be another source of error during LDF measurements. To reduce sensitivity to movement, a stabilising stent needs to be fabricated individually for each patient. Also, an accurate signal acquisition requires a fixed and constant position, minimising involuntary patient movement. The acquisition time of 3 min is used most frequently, especially due to the indications of the manufacturer [101-103]. This time interval causes a real discomfort to the patient, as he has to keep still during the test.

LDF could not be used through restorations to assess dental pulp blood flow. It is highly important that investigated teeth must be isolated in order to avoid laser light interacting with gingival blood flow. The assessments might be also susceptible to environmental and technique related factors [76]. In this case, a stabilising stent is the optimal solution helping to ensure that the tooth is always tested on the same part of the crown.

Another obvious obstacle for common use of this technology could be costs of commercially available equipment and computer technology storing and comparing the recorded data.

### Ultrasonic flowmeters

The basic function of ultrasonic flow meters (UFM) is to emit ultrasound and to detect reflected ultrasound [104], which permits to measure the velocity of a fluid and to calculate volume flow. The Doppler principle states that the frequency of the echo reflected from a moving target, such as red blood cells, will be different from the incident frequency [105]. Flow patterns can be detected by UFM from any accessible vessel, for instance, from the skin or oral mucosa surface.

Ultrasound is sound with a frequency that is higher than 20 kHz. In medical imaging, utilised ultrasound frequencies mainly range between 1 and 40 MHz. The transmission through air of such high frequencies is impossible, but they can satisfactorily pass through solid or fluid materials [106].

Every flowmeter has a probe consisting of piezoelectric crystal, which generates the ultrasound beam. A second crystal, slightly separated from the first one, detects the reflected ultrasound. It means that every ultrasonic transducer has a dual function as both transmitter and receiver of ultrasound. The probe is applied to the skin, and a specialised ultrasonic gel is used to conduct ultrasound. A signal produced by an ultrasonic transducer usually consists of a pulse of a few microseconds with a certain centre frequency. Part of this signal extends through the target tissue, part is reflected by macroscopic tissue structures, part is absorbed by tissue and part is scattered by structures in the tissue smaller than the acoustic wavelength [106]. The ultrasound is translated into audible sound, which allows hearing the pulsations in the vessel. Since the change in frequency is related to velocity, this can also be translated into vessel calibre [104].

The UFM technique was originally proposed by Satomura, Matsubara and Yoshioka (in 1956) for the physical measurement of minor vibrations [107]. In 1960, Satomura and Kaneko first described instantaneous changes in blood flow in human peripheral arteries using ultrasound blood-rheograph based on the Doppler effect. Later, Strandness, McCutcheon and Rushmer (in 1966) popularised transcutaneous flow detection for studying peripheral vascular problems [108].

### Innovative approach to measure blood flow by ultrasonic flowmeter

The UFM technique in dentistry is a well established and commonly used diagnostic tool in Russia and some other countries. Many researchers proved its useful role in prevention and early treatment of periodontal lesions [109-113].

Doppler ultrasonic flowmeter has the following advantages:

•Evaluation of blood in a limited gingival area (diameter of transducer is 1.5 mm);

•Metal constructions in the oral cavity are not limitations or contradictions for investigation of blood flow in the gingiva or tooth pulp;

•Investigation of a pulp blood flow;

•Possibility to detect blood flow in hard-to-access areas;

•Minimal time from measurement to obtain the results;

•Method is well tolerated by patients;

•Investigation could be repeated any number of times that allows controlling microcirculation changes over time.

There are several standardisation requirements for the UFM technique:

1. Patient should be at rest; there should be lack of physical activity before investigation,

2. During measurements, patient should be laying or sitting.

3. There should be comfortable temperature in the room (20°C–22°C).

4. It is forbidden to smoke or chew, in particular, a gum before measurements.

5. Investigator should not be pressured on the transducer not getting spurious result.

6. During measurements, the transducer should be placed at the same position.

**Figure 1:**
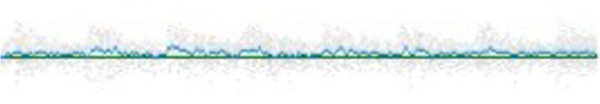
Doppler pulse curves with colour spectrum.

During ultrasonic blood flow investigation, the ultrasonic gel provides contact between the transducer and oral mucosa. A transducer 20–25 MHz is used to characterise periodontal blood flow. The ultrasound penetrates tissues at the depth of 0.8 сm. It is possible to control the position of transducer by sound and visual signals. It also helps to find and verify different types of blood vessels. For example, the arteries' pulsations are heard as continuously rising and falling, while the veins' sound is similar to that of the surf. The sound obtained from microcirculation is characterised as weak amplitude fluctuations on background of sea noise. The Doppler signal is processed in a computer and is displayed as pulse curves with colour spectrum called dopplerogramms (Figure 1).

Dopplerogramms help to visually determine the velocity of blood flow. It is known that the fastest erythrocytes are moving in the centre of the blood vessel. On a dopplerogramm, the fastest blood particles have a darker colour and could be seen at the rim of the curve in a distance from the baseline while the slowest ones are in the middle of the curve near the baseline. The programme also indicates the direction of blood flow: to transducer (‘+’, upper part of the baseline) or from transducer (‘−’, lower part of the baseline).

**Figure 2:**
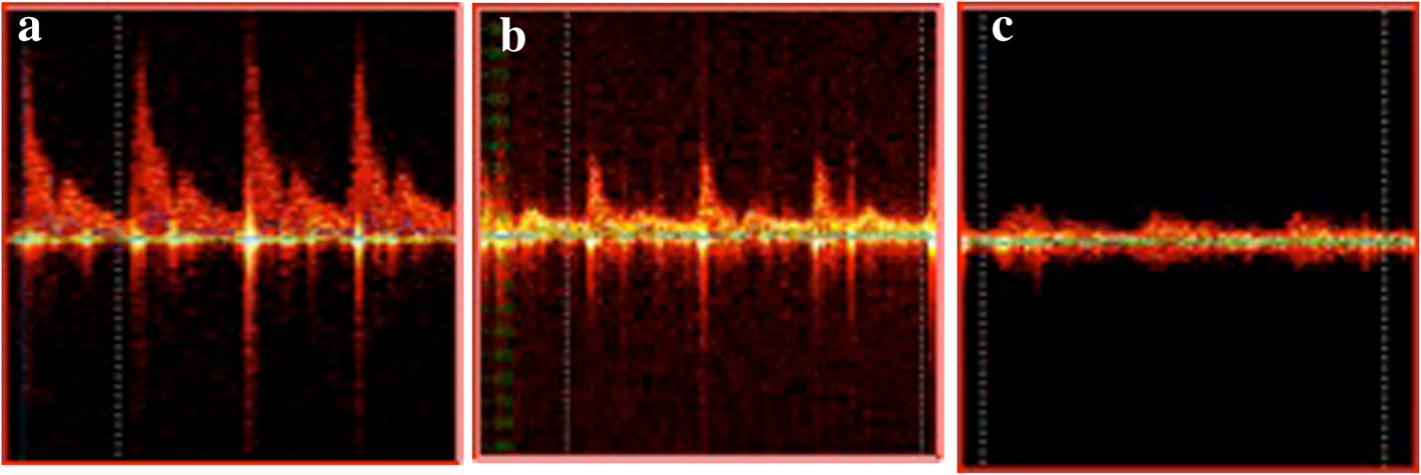
Ultrasonic Doppler signal obtained from (a) an artery, (b) arterioles, and (c) microcirculation.

Computer analysis of Doppler pulse curves provides information about the linear (systolic, mean, diastolic) and the volume velocity values of blood flow in the examined area. Qualitative and quantitative assessment of the blood flow is possible. The qualitative characteristic of the Doppler curve varies depending on the type and diameter of the vessel. Microcirculation (mixed blood flow) is characterised by pulse curves with colour spectrum with no sharp peaks (Figure 2).

UFM has several advantages in comparison with Doppler laser flowmetry [114]:

1. Audible and visual control of the position of transducer

2. Ability to determine a type of a blood vessel (artery, vein) by analysing the blood flow curve

3. Ability to analyse the distribution of the blood cells with different velocities in the investigating vessel

4. Detection of the direction of the blood flow

## Conclusions

Overall, laser and ultrasonic flowmeters are valuable tools in periodontal diagnosis and management. They might be especially useful for the prediction and prevention of periodontal diseases as well as for the management of gingivitis and periodontitis. However, they provide different information about blood flow in the investigated areas. That is why they are recommended to be used in combination for the prediction and prevention of periodontal lesions to provide more detailed and accurate data.

## Abbreviations

LDF: Laser Doppler flowmetry; UFM: Ultrasonic flowmetry.

## Competing interests

Both authors declare that they have no competing interests.

## Authors’ contributions

LO conceived the concept and idea of this article, participated in the collection of references, formed the thought line and outline of this article, coordinated, guided, led and corresponded the writing and heavily revised the entire manuscript. AB participated in the collection of references, constructed Figures 1 and 2, drafted most parts of the initial manuscript and participated in its revision. Both authors read and approved the final manuscript.

## Authors’ information

LO is a professor of dentistry, the President of the City Periodontal Center ‘PAKS’ (Saint-Petersburg, Russia), the Head of the Therapeutic Dentistry Department of the St. Petersburg State Medical University named after I.P. Pavlov, Vice-President of the St. Petersburg Dental Association, a member of the Scientific and Dissertation Council of the Pavlov Medical University, member of the Peter's Academy of Arts and Sciences, member of the National Academy of Esthetic Dentistry (Dental Association of Russia, Moscow) and Vice-President of the Periodontology section of the Russian Dental Association. LO is a leading specialist in the field of periodontics, immunology and dental care as well as in undergraduate and postgraduate training of medical service providers in St. Petersburg and other regions of the Russian Federation. AB is the assistant of the Therapeutic Dentistry Department at the I.P. Pavlov Saint-Petersburg State Medical University; her PhD was supervised by Professor Liudmila Yu Orekhova; she focuses on the studies of oral conditions, in particular, periodontal lesions, and microcirculation disturbances in oral cavity.
